# Subolesin expression in response to pathogen infection in ticks

**DOI:** 10.1186/1471-2172-11-7

**Published:** 2010-02-19

**Authors:** Zorica Zivkovic, Alessandra Torina, Ruchira Mitra, Angela Alongi, Salvatore Scimeca, Katherine M Kocan, Ruth C Galindo, Consuelo Almazán, Edmour F Blouin, Margarita Villar, Ard M Nijhof, Rinosh Mani, Giuseppa La Barbera, Santo Caracappa, Frans Jongejan, José de la Fuente

**Affiliations:** 1 Department of Infectious Diseases and Immunology, Utrecht Centre for Tick-borne Diseases (UCTD), Faculty of Veterinary Medicine, Utrecht University, Yalelaan 1, 3584CL, Utrecht, The Netherlands; 2 Intituto Zooprofilattico Sperimentale della Sicilia, Via G. Marinuzzi n°3, 90129 Palermo, Sicily, Italy; 3 Department of Veterinary Pathobiology Center for Veterinary Health Sciences, Oklahoma State University, Stillwater, OK 74078, USA; 4 Instituto de Investigación en Recursos Cinegéticos IREC (CSIC-UCLM-JCCM), Ronda de Toledo s/n, 13005 Ciudad Real, Spain; 5 Facultad de Medicina Veterinaria y Zootecnia, Universidad Autónoma de Tamaulipas, Km. 5 carretera Victoria-Mante, CP 87000 Ciudad Victoria, Tamaulipas, Mexico; 6 Department of Veterinary Tropical Diseases, Faculty of Veterinary Science, University of Pretoria, Private Bag X04, 0110, Onderstepoort, South Africa

## Abstract

**Background:**

Ticks (Acari: Ixodidae) are vectors of pathogens worldwide that cause diseases in humans and animals. Ticks and pathogens have co-evolved molecular mechanisms that contribute to their mutual development and survival. Subolesin was discovered as a tick protective antigen and was subsequently shown to be similar in structure and function to akirins, an evolutionarily conserved group of proteins in insects and vertebrates that controls NF-kB-dependent and independent expression of innate immune response genes. The objective of this study was to investigate subolesin expression in several tick species infected with a variety of pathogens and to determine the effect of subolesin gene knockdown on pathogen infection. In the first experiment, subolesin expression was characterized in ticks experimentally infected with the cattle pathogen, *Anaplasma marginale*. Subolesin expression was then characterized in questing or feeding adult ticks confirmed to be infected with *Anaplasma*, *Ehrlichia*, *Rickettsia*, *Babesia *or *Theileria *spp. Finally, the effect of subolesin knockdown by RNA interference (RNAi) on tick infection was analyzed in *Dermacentor variabilis *males exposed to various pathogens by capillary feeding (CF).

**Results:**

Subolesin expression increased with pathogen infection in the salivary glands but not in the guts of tick vector species infected with *A. marginale*. When analyzed in whole ticks, subolesin expression varied between tick species and in response to different pathogens. As reported previously, subolesin knockdown in *D. variabilis *infected with *A. marginale *and other tick-borne pathogens resulted in lower infection levels, while infection with *Francisella tularensis *increased in ticks after RNAi. When non-tick-borne pathogens were fed to ticks by CF, subolesin RNAi did not affect or resulted in lower infection levels in ticks. However, subolesin expression was upregulated in *D. variabilis *exposed to *Escherichia coli*, suggesting that although this pathogen may induce subolesin expression in ticks, silencing of this molecule reduced bacterial multiplication by a presently unknown mechanism.

**Conclusions:**

Subolesin expression in infected ticks suggested that subolesin may be functionally important for tick innate immunity to pathogens, as has been reported for the akirins. However, subolesin expression and consequently subolesin-mediated innate immunity varied with the pathogen and tick tissue. Subolesin may plays a role in tick innate immunity in the salivary glands by limiting pathogen infection levels, but activates innate immunity only for some pathogen in the guts and other tissues. In addition, these results provided additional support for the role of subolesin in other molecular pathways including those required for tissue development and function and for pathogen infection and multiplication in ticks. Consequently, RNAi experiments demonstrated that subolesin knockdown in ticks may affect pathogen infection directly by reducing tick innate immunity that results in higher infection levels and indirectly by affecting tissue structure and function and the expression of genes that interfere with pathogen infection and multiplication. The impact of the direct or indirect effects of subolesin knockdown on pathogen infection may depend on several factors including specific tick-pathogen molecular interactions, pathogen life cycle in the tick and unknown mechanisms affected by subolesin function in the control of global gene expression in ticks.

## Background

Ticks transmit pathogens of the genera *Anaplasma*, *Ehrlichia*, *Rickettsia*, *Babesia *and *Theileria *that impact both human and animal health [1-3]. Of these tick-borne pathogens, *Anaplasma marginale *causes the economically important cattle disease, bovine anaplasmosis [2]. Worldwide, *A. marginale *is vectored by tick species of the genera *Dermacentor *and *Rhipicephalus *[1-3]. The developmental cycle of *A. marginale*, which is presently the most completely characterized rickettsial cycle in ticks, is complex and coordinated with tick feeding cycle [4-6]. Ticks become infected with *A. marginale *when they ingest infected bovine erythrocytes in the bloodmeal, and the first sites of infection are in gut and Malpighian tubule cells. After a second tick feeding, *A. marginale *infects and develops in salivary glands, the site of transmission to the vertebrate host.

The ticks and the pathogens that they transmit have co-evolved molecular interactions involving genetic traits of both the tick and the pathogen that mediate their development and survival [7]. Recent studies have shown that pathogen infection modifies the expression of subolesin and other tick genes [7-11]. Tick subolesin was discovered as a tick protective antigen in *Ixodes scapularis *[12]. Subolesin was shown by RNAi gene knockdown and immunization trials using the recombinant protein to protect hosts against tick infestations, reduce tick survival and reproduction, cause degeneration of guts, salivary glands, reproductive tissues and embryos and to decrease the vector capacity of ticks for *A. marginale *and *A. phagocytophilum *[8,13-18]. In addition, subolesin was shown to be similar in structure and function to insect and vertebrate akirins which control NF-kB-dependent and independent gene expression that impact innate immunity [19-22]. Based on the proposed function for tick subolesin, this molecule would be involved in the initial host innate immune response to pathogen infection. However, subolesin expression and its role in tick innate immunity to pathogen infection have not been reported.

The objective of this study was to investigate subolesin expression in several tick species infected with a variety of pathogens and to determine the effect of subolesin gene knockdown on pathogen infection.

## Results

### Expression of subolesin in tick vectors experimentally infected with *A. marginale*

**Figure 1:**
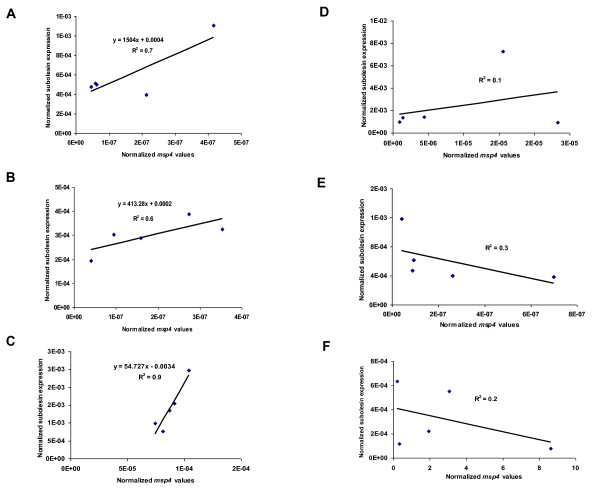
**Correlation between subolesin expression and *A. marginale *infection levels in *D. variabilis *male guts and salivary glands**. RNA was extracted from guts collected after acquisition feeding (D-E) and salivary glands collected after transmission feeding (A-C) in 5 pools of 10 ticks each of *D. variabilis *(A and D), *D. andersoni *(B and E) and *R. sanguineus *(C and F) male ticks experimentally infected with *A. marginale*. Subolesin and *msp4 *mRNA levels were analyzed by real-time RT-PCR and normalized against tick 16S rRNA using the comparative Ct method [9,32]. Regression analyses were conducted in Microsoft Excel to compare normalized *A. marginale msp4 *and subolesin mRNA levels.

Subolesin expression was analyzed in the tick vector species, *D. variabilis*, *D. andersoni*, *D. reticulatus*, *R. sanguineus*, *R. microplus *and *R. annulatus *experimentally infected with *A. marginale*. Characterization of subolesin expression in guts and salivary glands was done in *D. variabilis*, *D. andersoni *and *R. sanguineus*. Differences in subolesin expression were observed between guts and salivary glands when correlated with *A. marginale *infection in *D. variabilis*, *D. andersoni *and *R. sanguineus *(Figures 1A-F). While subolesin expression in salivary glands correlated positively with pathogen infection in all three tick species (correlation coefficient, R^2 ^= 0.7, 0.6 and 0.9, for *D. variabilis*, *D. andersoni *and *R. sanguineus*, respectively; Figures 1A-C), a correlation was not found in guts (R^2 ^= 0.1, 0.3 and 0.2, respectively; Figures 1D-F). Interestingly, as shown by differences in the linear correlation slope, the increase in pathogen infection levels resulted in larger variations in subolesin expression in *R. sanguineus *(Figure 1C) as compared to *Dermacentor *spp. (Figures 1A and 1B).

**Figure 2:**
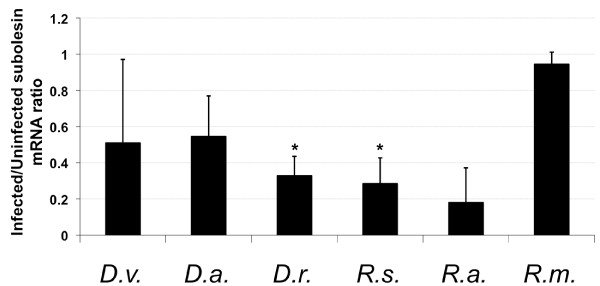
**Subolesin expression in tick vector species experimentally infected with *A. marginale***. Subolesin expression was characterized in *D. variabilis (D.v*.), *D. andersoni (D.a*.), *D. reticulatus (D.r*.), *R. sanguineus (R.s*.), *R. annulatus (R.a*.) and *R. microplus (R.m*.) whole ticks after transmission feeding (5 pools of 10 ticks each). Subolesin mRNA levels were analyzed by real-time RT-PCR and normalized against tick 16S rRNA using the comparative Ct method [9,32]. The graph depicts the infected to uninfected subolesin mRNA ratio (± SD) calculated by dividing normalized subolesin mRNA levels in infected ticks by the average of the normalized subolesin mRNA level in uninfected control ticks (N = 20). Normalized subolesin mRNA levels were compared between infected and uninfected ticks by Student's t-Test (*P < 0.05).

When subolesin expression was analyzed in whole ticks, differences were observed in response to *A. marginale *infection between tick species, but in all cases subolesin levels remained unchanged (4 of 6 species analyzed) or were significantly lower in infected ticks than in the uninfected controls (2 of 6 species analyzed) (Figure 2). However, notable tick-to-tick variation in subolesin expression was also observed (Figure 2).

### Subolesin expression in questing and feeding adult ticks naturally infected with *Anaplasma*, *Ehrlichia*, *Rickettsia*, *Babesia *or *Theileria *species

To characterize subolesin expression in ticks naturally infected with different pathogens, questing and feeding adult ticks were collected and analyzed for pathogen infection. The ticks were found to be infected with various pathogens: *R. sanguineus *and *D. marginatus *were infected with *Rickettsia conorii*; *R. bursa *was infected with *Theileria annulata*; *Hyalomma lusitanicum *was infected with *Babesia bigemina*; *Hyalomma marginatum marginatum *was infected with *Theileria buffeli*; *R. sanguineus *was infected with *Ehrlichia canis*; and *R. turanicus *and *R. bursa *were infected with *A. ovis *(Table 1).

**Table 1:** Adult ticks naturally infected with *Anaplasma*, *Ehrlichia*, *Rickettsia*, *Babesia *or *Theileria *species.

Tick species (N)	Sex	Collection	Pathogen infection
*R. sanguineus *(3)	female	questing	*R. conorii*

*D. marginatus *(3)	female	questing	*R. conorii*

*R. bursa *(9)	female	sheep	*T. annulata*

*H. lusitanicum *(5)	male	questing	*B. bigemina*

*H. m. marginatum *(8)	male	cattle	*T. buffeli*

*R. sanguineus *(2)	female	dog	*E. canis*

*R. turanicus *(2)	female	sheep	*A. ovis*

*R. bursa *(3)	female	sheep	*A. ovis*

Questing and feeding adult ticks were collected in Sicilian farms and analyzed for pathogen infection by PCR or RLB. To define pathogen species infecting ticks, PCR and sequence analysis of cloned amplicons were performed for *Anaplasma*, *Ehrlichia *and *Rickettsia *spp. For *Theileria *and *Babesia *spp., RLB results were confirmed at the species level. For analysis of subolesin expression, sex and collection-matching uninfected controls were used. Uninfected ticks were negative for all pathogens analyzed.

**Figure 3:**
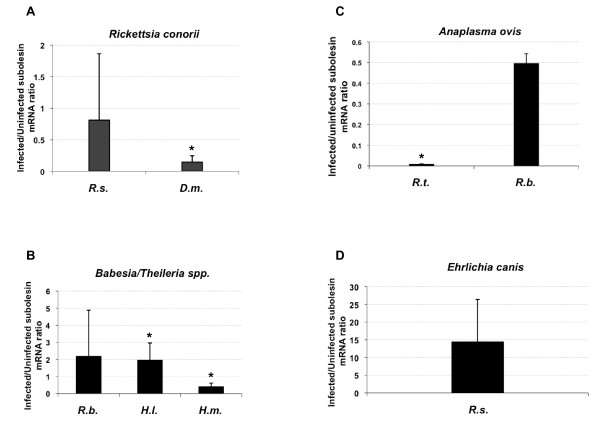
**Subolesin expression in questing or feeding adult ticks naturally infected with different pathogens**. Subolesin expression was characterized in *R. sanguineus (R.s*.) and *D. marginatus (D.m*.) infected with *R. conorii *(A), *R. bursa (R.b*.), *H. lusitanicum (H.l*.) and *H. m. marginatum (H.m*.) infected with *T. annulata*, *B. bigemina *and *T. buffeli*, respectively (B), *R. turanicus *and *R. bursa *infected with *A. ovis *(C) and *R. sanguineus *infected with *E. canis *(D). In all cases, sex and collection-matching groups of uninfected tick samples were analyzed for comparison. Subolesin mRNA levels were analyzed by real-time RT-PCR and normalized against tick 16S rRNA using the comparative Ct method [9,32]. The graph depicts the infected to uninfected subolesin mRNA ratio (± SD) calculated by dividing normalized subolesin mRNA levels in infected ticks by the average of the normalized subolesin mRNA level in uninfected control ticks. Normalized subolesin mRNA levels were compared between infected and uninfected ticks by Student's t-Test (*P < 0.05).

Subolesin mRNA levels were analyzed in infected ticks and in sex and collection-matched uninfected controls. Under natural infection conditions, differences in subolesin expression were observed between tick species in response to different pathogens (Figures 3A-D). However, similar to ticks experimentally infected with *A. marginale*, subolesin levels remained unchanged or were lower in infected ticks as compared with uninfected controls (Figures 3A-D) with the exception of *H. lusitanicum *infected with *B. bigemina *(Figure 3B). Tick-to-tick variations in subolesin expression were also observed as shown previously in ticks experimentally infected with *A. marginale *(Figures 3A-D). When analysis was conducted in the same tick species infected with different pathogens, *R. sanguineus *infected with *R. conorii *or *E. canis *(Figures 3A and 3D) and *R. bursa *infected with *T. annulata *or *A. ovis *(Figures 3B and 3C), subolesin expression levels did not differ with the pathogen and were similar between infected and uninfected ticks.

### Effect of subolesin knockdown on the tick response to pathogen infection

The results of subolesin expression studies in response to pathogen infection suggested a role for this molecule in tick innate immunity, at least in salivary glands and in whole ticks in response to some pathogens.

To define the role of subolesin in tick innate immunity, the effect of subolesin gene knockdown was analyzed in *D. variabilis *males capillary fed Gram-positive and Gram-negative bacteria and the yeast, *Pichia pastoris *(Table 2). The results demonstrated that subolesin knockdown after RNAi was effective with an average of 55-99% gene silencing (Table 2). The effect of subolesin knockdown on tick infection levels varied among pathogens (Table 2). While *Francisella tularensis *infection levels were higher in subolesin-silenced ticks when compared to controls, the *A. marginale*, *A. phagocytophilum*, *Ehrlichia canis *and *Escherichia coli *levels were lower. *Bacillus subtilis *and *P. pastoris *infection levels were not significantly different between subolesin-silenced and control ticks.

**Table 2:** Experimental conditions and results of *D. variabilis *subolesin RNAi and CF with different pathogens.

Pathogen (isolate/strain)	Inoculum	CF tickmeal	Subolesin expression silencing (%)^**a**^	Tick infection ratio (Subolesin/Rs86)^**b**^
*A. marginale* (Oklahoma, OK [33])	4.3% infected erythrocytes	Blood from splenectomized calves experimentally infected with isolate stabilate	89 ± 17*	0.85 ± 0.09*

*A. marginale* (Okeechobee, FL [33])	3.3% infected erythrocytes	Blood from splenectomized calves experimentally infected with isolate stabilate	55 ± 32*	0.83 ± 0.10*

*A. marginale* (Bison) [33]	7.4% infected erythrocytes	Blood from splenectomized calves experimentally infected with isolate stabilate	86 ± 17*	0.95 ± 0.10*

*A. phagocytophilum* (NY18) [34]	50% infected cells	ISE6 cultured tick cells in L15B with 10% FBS	92 ± 14*	0.91 ± 0.09*

*F. tularensis* (Live Vaccine Strain LVS; ATCC 29684)	10^7 ^CFU/ml	DMEM with 10% FBS	99 ± 2*	1.74 ± 0.86*

*E. canis* (Ebony)	2% infected cells	DH82 cultured dog cells in DMEM with 10% FBS	94 ± 11*	0.89 ± 0.16*

*E. coli* (JM109; Promega)	10^7 ^CFU/ml	DMEM with 10% FBS	97 ± 3*	0.92 ± 0.07*

*B. subtilis* (culture 125-1 kindly supplied by H. Evers)	10^7 ^CFU/ml	DMEM with 10% FBS	71 ± 21*	0.65 ± 0.58

*P. pastoris* (X33; Invitrogen)	10^6 ^CFU/ml	YPD	80 ± 16*	0.60 ± 0.31

^**a**^Subolesin mRNA levels were determined by real-time RT-PCR and normalized against tick 16S rRNA using the comparative Ct method. Percent subolesin expression silencing was calculated in subolesin dsRNA-injected ticks with respect to control ticks injected with the unrelated Rs86 dsRNA and expressed as average ± SD. Subolesin normalized Ct values were compared between subolesin dsRNA and control Rs86 dsRNA injected ticks by Student's t-test (*P < 0.05).

^**b**^Infection levels were determined by real-time PCR using pathogen-specific gene sequences and normalizing against tick 16S rRNA using the comparative Ct method. Tick infection ratio was calculated as subolesin dsRNA to average control Rs86 dsRNA injected ticks normalized Ct values and expressed as average ± SD. Pathogen-specific gene normalized Ct values were compared between subolesin dsRNA and control Rs86 dsRNA injected ticks by Student's t-test (*P < 0.05).

Abbreviations: CF, capillary feeding; CFU, colony forming units; L15B, modification of Leibovitz's L15 medium containing additional glucose, amino acids, vitamins and trace minerals (Sigma-Aldrich, St Louis, MO, USA); FBS, fetal bovine serum (Sigma); DMEM, Dulbecco's Modified Eagle Medium (Gibco, Invitrogen, Carlsbad, CA, USA); YPD, Yeast Extract Peptone Dextrose medium (10 g/l yeast extract, 20 g/l peptone, 20 g/l glucose) (Sigma).

**Figure 4:**
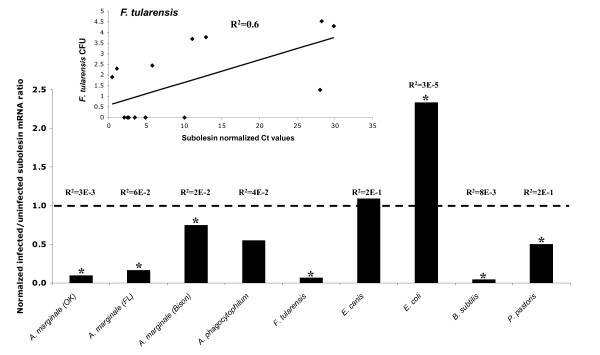
**Subolesin expression in *D. variabilis *male ticks infected with different pathogens by capillary feeding (CF)**. Subolesin expression levels were compared between ticks injected with control Rs86 dsRNA and then fed pathogen-infected or plain media by CF (N = 27-29). Whole individual ticks were dissected and used for DNA/RNA extraction to determine pathogen infection levels by real-time PCR and subolesin mRNA levels by real-time RT-PCR after normalization against tick 16S rRNA using the comparative Ct method [9,32]. The graph depicts the infected to uninfected subolesin mRNA ratio (± SD) calculated by dividing normalized subolesin mRNA level in infected ticks by the average of the normalized subolesin mRNA level in uninfected control ticks. Normalized subolesin mRNA levels were compared between infected and uninfected ticks by Student's t-Test (*P < 0.05). Regression analyses were conducted in Microsoft Excel to compare normalized pathogen infection levels and subolesin mRNA levels. Regression coefficients are shown for all groups. The correlation graph is shown in the insert for *F. tularensis*, the only group in which a positive correlation was found between subolesin expression and pathogen infection levels.

**Figure 5:**
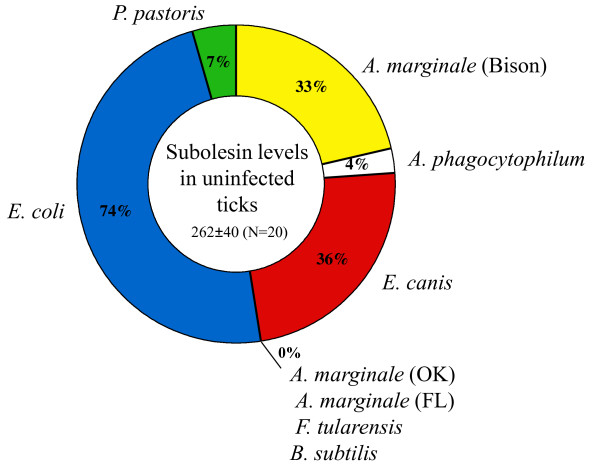
**Tick-to-tick variations in subolesin expression in response to pathogen infection**. The graph depicts the percent of infected male *D. variabilis *ticks that showed normalized subolesin mRNA levels higher than the average expression level in uninfected ticks. In all experiments, 27-29 infected ticks were analyzed. For experimental details see figure 4 legend.

To characterize the effect of pathogen infection by CF on subolesin expression, subolesin mRNA levels were compared between ticks injected with control dsRNA and then fed pathogen-infected or plain media by CF (Figure 4). The results demonstrated that, with the exception of the *E. coli*-fed ticks, subolesin levels remained unchanged or were lower in infected ticks. However, when subolesin expression was analyzed in individual ticks, some ticks in groups infected with *E. coli*, *E. canis*, *A. marginale *(Bison), *P. pastoris *and *A. phagocytophilum *had subolesin mRNA levels higher than the controls (Figure 5). This result explained the tick-to-tick variation observed in previous experiments with experimentally and naturally infected ticks and suggested that other factors affected subolesin expression independent of infection levels because subolesin expression only correlated positively with pathogen infection levels in *F. tularensis*-infected ticks (Figure 4 insert).

## Discussion

Differential expression of subolesin in *Anaplasma*-infected tick guts and salivary glands and cultured tick cells was reported previously [7,9,10]. In these experiments, subolesin expression was significantly upregulated in *D. variabilis *salivary glands and IDE8 tick cells but not in *D. variabilis *guts and *R. microplus *salivary glands in response to infection with *A. marginale *[9]. In contrast to *A. marginale*, subolesin expression in *A. phagocytophilum*-infected *I. scapularis *nymphs was significantly downregulated and remained unchanged in infected ISE6 cultured tick cells [9]. The differences in expression patterns between *A. marginale *and *A. phagocytophilum *infected cultured tick cells were also recently demonstrated for other genes [11]. In addition, functional analysis by subolesin RNAi demonstrated that *A. marginale *infection levels were reduced in *D. variabilis *salivary glands and IDE8 tick cells after gene knockdown [8-10]. Subolesin knockdown affected *A. marginale *development in *D. variabilis *by affecting rickettsial development and infection levels in different tissues [10]. Interestingly, salivary gland infections were not observed in these subolesin-silenced ticks, raising the question of whether they would have been able to transmit *A. marginale *[10]. Additionally, the function of subolesin was recently suggested to be similar to insect and vertebrate akirins in the control of NF-kB-dependent and independent gene expression in ticks [20,21]. These results suggested that subolesin expression would likely be affected by pathogen infection and to have a role on tick innate immunity, a hypothesis that was tested in the experiments reported herein.

Results reported herein (Table 1) further confirmed subolesin upregulation in salivary glands of *A. marginale*-infected ticks. However, *A. marginale *infection did not affect subolesin expression in the gut of infected ticks. When subolesin expression was analyzed in whole ticks infected with various pathogens, expression levels remained generally unchanged or were lower in infected ticks. This result suggested that while subolesin expression may be upregulated in salivary glands, expression may not be affected or even decreased in other tissues of infected ticks. The overall effect of pathogen infection on subolesin expression in whole ticks may be different from that observed in isolated tissues and reinforces the role that different tissues play in pathogen infection and multiplication in ticks [10,23]. These results were similar to those obtained in *I. scapularis *nymphs infected with *A. phagocytophilum *but demonstrated differences in tick response to other pathogens, as illustrated by subolesin upregulation in *H. lusitanicum *infected with *B. bigemina *[9]. However, the results with naturally infected ticks should be taken with caution due to the small number of ticks analyzed. Furthermore, although naturally infected ticks were analyzed for the presence of the most prevalent tick-borne pathogens in Sicily, the infection with other pathogens not considered in these studies could affect subolesin expression levels in ticks [24]. However, it is likely that these pathogens would be present in both groups of ticks considered in the study, "infected" and "uninfected", therefore rendering no differences in subolesin mRNA levels.

As demonstrated herein, subolesin may play a role in tick innate immunity in salivary glands but not in the gut. Since the gut is the first tick barrier to pathogen infection [2], subolesin may not be involved in protecting ticks against *A. marginale *infection because of mechanisms that have co-evolved between the pathogen and the tick vector to support pathogen transmission while insuring tick survival [25]. However, subolesin may function in the salivary glands to limit pathogen infection to levels that are not detrimental for ticks. The differential role of subolesin in tick gut and salivary gland cells was further demonstrated by RNAi experiments [7,9]. Gene knockdown may not affect *A. marginale *infection levels in tick guts because subolesin may not be involved in innate immunity in this tissue. The decrease in *A. marginale *infection levels in the salivary glands of subolesin-silenced *D. variabilis *may not be related to innate immune response but may be due to suppression of other genes regulated by subolesin and required for pathogen infection and multiplication [7,19,20] and/or involved in salivary gland function [10,15]. This effect is most likely not relevant in the gut perhaps because gene silencing was shown to occur 3 days after injection of subolesin dsRNA [15], when gut cells may have already become infected with *A. marginale*.

Recently, we studied the role of *D. variabilis *defensin, varisin, in tick innate immunity to *A. marginale *[26]. Silencing of varisin occurred in tick hemocytes, midguts and salivary glands after RNAi. Varisin knockdown did not increase *A. marginale *infections, which actually were significantly reduced in the varisin-silenced ticks. However, *A. marginale *colonies were morphologically abnormal in varisin-silenced ticks when compared with the controls and some ticks had systemic infections with a yeast-like microbe that may have resulted from varisin RNAi. These results suggested that tick-pathogen interactions may have evolved in natural tick vector species to prevent innate immunity mechanisms against the vectored pathogen and to limit infection with non-tick vectored microorganisms.

The effect of subolesin knockdown in ticks on infection with tick-borne and non-tick-borne organisms was tested in *D. variabilis *using RNAi and CF. In these experiments, infection levels of the tick-borne pathogens, *A. marginale*, *A. phagocytophilum *and *E. canis *were lower in subolesin-silenced ticks. In contrast, *F. tularensis *infections were higher after subolesin RNAi and CF. These results could be explained in several ways: (1) as previously discussed, tick-pathogen interactions may have evolved in the natural tick vector species to prevent innate immunity against tick-borne pathogens; (2) the life cycle of pathogens in ticks varies and may be accompanied by different impacts of subolesin expression; (3) although *D. variabilis *has been demonstrated to be a vector for *F. tularensis *[27], as shown herein, subolesin function in innate immunity could differ among pathogens; (4) subolesin control of gene expression in ticks may include the expression of genes crucial for tissue structure and function and pathogen infection and multiplication [8-10,19,20]; (5) as suggested by CF experiments, unknown individual factors may affect tick subolesin expression and the capacity of ticks to mount an effective subolesin-mediated innate immune response to pathogen infection; and (6) pathogen infection by CF may differ from natural conditions and thus change the role of subolesin in pathogen infection and development. For example, *A. marginale *infection and multiplication in capillary fed ticks occurred only in the gut [28], thus differing from the natural life cycle.

For non-tick-borne pathogens, *E. coli *and *P. pastoris*, RNAi experiments suggested that subolesin did not have an effect on infection, at least after CF. However, subolesin expression was upregulated in *D. variabilis *exposed to *E. coli*, suggesting that although this pathogen may induce subolesin expression in ticks, silencing of this molecule reduced bacterial multiplication by a presently unknown mechanism. As discussed previously, tick-to-tick variations in subolesin expression in response to pathogen infection occurred for *E. coli *and *P. pastoris*, again suggesting that subolesin may play a role in tick innate immunity against these pathogens but this effect could be affected by unknown individual factors.

Previously, Goto et al. [21] demonstrated that akirin or relish knockdown in flies resulted in lower survival rates after *Agrobacterium tumefasciens *infection when compared to controls. The experiments conducted in ticks were not designed to study the effect of subolesin knockdown on infected tick survival. However, in agreement with lower infection levels in ticks after subolesin silencing, we did not observe an increase in tick mortality after experimental infection with *A. marginale *and other pathogens by CF. The discrepancy between the results obtained for flies and ticks after akirin/subolesin knockdown and pathogen infection could be explained by: (1) interactions that resulted from the tick-pathogen co-evolution, which are not present in *A. tumefasciens*-infected flies, (2) the limitations of CF to mimic natural tick feeding and infection conditions, (3) differences in the function of subolesin when compared to that of akirin in insects or (4) a combination of these factors.

## Conclusions

These studies demonstrated that subolesin expression varies with pathogen infection in tick salivary glands and in the guts in response to some pathogens, thus suggesting a role of subolesin in tick innate immunity. Subolesin may activate innate immunity to certain pathogens in tick salivary glands, resulting in lower pathogen infection levels. This function may occur to a lesser extent in tick midguts and other tissues, although results in *H. lusitanicum *infected with *B. bigemina *suggest activation of innate immunity at the gut level in particular vector-pathogen systems (Figure 3B). Furthermore, previous studies have suggested a role of subolesin in different molecular pathways, including those involved in normal tick physiology and in pathogen infection and multiplication in ticks. Consequently, subolesin knockdown may affect pathogen infection in ticks directly by reducing innate immune responses resulting in higher infection levels and indirectly by affecting the expression of genes that interfere with tissue physiology and pathogen infection and multiplication.

## Methods

### Ticks

*D. variabilis*, *D. andersoni *and *R. sanguineus *male ticks were obtained from the Tick Rearing Facility, Department of Entomology and Plant Pathology, Oklahoma State University. Larvae and nymphs were fed on rabbits and adults were fed on sheep. The *R. annulatus *(Mercedes strain, Texas, USA) and *R. microplus *(Mozambique strain) ticks were obtained from laboratory colonies maintained on cattle at the University of Tamaulipas and the Utrecht Centre for Tick-Borne Diseases, University of Utrecht, The Netherlands, respectively. *D. reticulatus *ticks were also obtained from a laboratory colony at the tick rearing facility at the University of Utrecht. Larvae and nymphs were fed on rabbits and adults were fed on calves. Off-host ticks were maintained in a 12 hr light: 12 hr dark photoperiod at 22-25°C and 95% relative humidity. Animals were cared for in accordance with standards specified in the Guide for Care and Use of Laboratory Animals of each institution.

To obtain *A. marginale*-infected ticks, *D. variabilis*, *D. andersoni *and *R. sanguineus*, male ticks were allowed to acquisition feed (AF) for one week, during an ascending parasitemia, on a splenectomized calf experimentally-infected with the Virginia isolate of *A. marginale*. The ticks were then removed and maintained off-host for 4 days and then allowed to transmission feed (TF) for an additional week on an uninfected calf. *R. annulatus *larvae were allowed to feed on a calf naturally-infected with *A. marginale *in Tamaulipas, Mexico (approximately 4% rickettsemia during tick feeding) and collected as adults after 21 days of feeding. *R. microplus *larvae and *D. reticulatus *adult male ticks were allowed to feed on an intact calf experimentally infected with the Nigeria isolate of *A. marginale*. *R. microplus *males were collected after 21 days of feeding. *D. reticulatus *ticks were allowed to AF for 7 days, removed and maintained 5 days off-host and then allowed to TF for an additional week on the same infected calf. Uninfected ticks were allowed to feed in the same way on uninfected calves to serve as controls. Infection of ticks with *A. marginale *was determined by *msp4 *PCR [29]. Cattle were maintained according to approved protocols and under the supervision of the respective Institutional Animal Care and Use Committees.

Questing and feeding adult ticks were collected on 27 farms located in different Sicilian regions (Palermo, Enna, Messina, Siracusa and Trapani). A total of 678 ticks were collected and analyzed for this study. Of them, 29 were questing ticks and 649 were collected from cattle, sheep, goats or dogs. Ticks were identified using morphological keys for the Italian Ixodidae [30]. The ticks were incubated for three days in the laboratory prior to dissection and RNA/DNA extraction.

### Identification of pathogen infection in naturally infected ticks

DNA was extracted from individual whole tick samples using TriReagent (Sigma, St. Louis, MO, USA) following manufacturers recommendations. The DNA was resuspended in sterile distilled water and stored at -20°C until used. For the initial screening, PCR analyses for *Anaplasma*, *Ehrlichia *and *Rickettsia *spp. were performed as described previously [24] with 1 μl (0.1-10 ng) DNA using 10 pmol of each primer and the Ready-To-Go PCR beads (Amersham, Piscataway, NJ, USA). Reactions were performed in an automated DNA thermal cycler for 35 cycles. PCR products were electrophoresed on 1% agarose gels to check the size of amplified fragments by comparison to a DNA molecular weight marker (1 Kb DNA Ladder, Promega). Control reactions were done without the addition of DNA to the reaction to rule out contaminations during PCR. Reverse line blot (RLB) was used for detection of *Babesia/Theileria *spp. as described previously [31]. Uninfected ticks were confirmed to be negative for all pathogens analyzed.

To identify and confirm pathogens in ticks, PCR and sequence analysis of cloned amplicons were performed for *Anaplasma*, *Ehrlichia *and *Rickettsia *spp. Amplified fragments were resin purified (Promega), cloned into pGEM-T vector (Promega) and sequenced in an accredited service laboratory (BaseClear, Leiden, The Netherlands) using vector specific primers. The BLAST tool was used to search the NCBI databases in order to identify sequences reported previously with identity to sequences obtained herein. Gene sequences were deposited in the GenBank with accession numbers GQ857075-GQ857078.

### Gene expression analysis by real-time RT-PCR in experimentally and naturally infected ticks

Total RNA was extracted using TriReagent (Sigma) following manufacturers recommendations. In *D. variabilis*, *D. andersoni *and *R. sanguineus *male ticks experimentally infected with *A. marginale*, RNA was extracted from guts collected after AF and salivary glands collected after TF in 5 pools of 10 ticks each. *A. marginale *infection in tick guts and salivary glands was characterized by *msp4 *real-time RT-PCR as described previously [7]. Subolesin expression was characterized by real-time RT-PCR using species-specific oligonucleotide primers (Table 3) as described previously [9]. Subolesin levels were characterized in guts and salivary glands of *D. variabilis*, *D. andersoni *and *R. sanguineus *and in whole ticks experimentally infected with *A. marginale *after TF (5 pools of 10 ticks each) and in individual whole ticks naturally-infected with different pathogens. In all cases, matching groups of uninfected tick samples were analyzed concurrently for comparison. Real-time RT-PCR was done using the QuantiTec SYBR Green RT-PCR kit (Qiagen, Valencia, CA, USA) and a Bio-Rad iQ5 thermal cycler (Hercules, CA, USA) following manufacturer's recommendations. mRNA levels were normalized against tick 16S rRNA using the comparative Ct method [9,32]. Normalized subolesin mRNA levels were compared between infected and uninfected ticks by Student's t-Test (P = 0.05). Regression analyses were conducted in Microsoft Excel to compare normalized *A. marginale msp4 *and subolesin mRNA levels in the guts and salivary glands of *D. variabilis*, *D. andersoni *and *R. sanguineus *male ticks experimentally infected with *A. marginale*.

**Table 3:** Oligonucleotide primers and PCR conditions for the characterization of subolesin and pathogen-specific gene expression.

Gene description^**a**^	Upstream/downstream primer sequences (5'-3')	PCR annealing conditions
*D. variabilis *subolesin [9]	CCAGCCTCTGTTCACCTTTC CCGCTTCTGAATTTGGTCAT	54°C, 30 sec

*R. microplus *subolesin [9]	CACAGTCCGAGTGGCAGAT GATGCACTGGTGACGAGAGA	55°C, 30 sec

*A. marginale msp4 *[29]	GGGAGCTCCTATGAATTACAGAGAATTGTTTAC CCGGATCCTTAGCTGAACAGGAATCTTGC	60°C, 1 min

*A. phagocytophilum msp4 *[9]	GACGTGCTGCACACAGATTT CTCATCAAATAGCCCGTGGT	54°C, 1 min

*E. canis *16S (M73221)	GTGGCAGACGGGTGAGTAAT GCTGATCGTCCTCTCAGACC	57°C, 30 sec

*B. subtilis dal *[35]	AATTGAAAGGGACCGACATC- TTAATGGTTTCGAGCCTTCC	59°C, 30 sec

*E. coli dxs *[36]	CGAGAAACTGGCGATCCTTA CTTCATCAAGCGGTTTCACA	60°C, 30 sec

*P. pastoris *CTA 1 (AB472085)	CCTGAAGGACGCCAATATGT GCTTTCCAGCCTCTTCATTG	57°C, 30 sec

Tick 16S rRNA [9]	GACAAGAAGACCCTA ATCCAACATCGAGGT	42°C, 30 sec

^a^When published, references are shown for oligonucleotide sequences. When designed for this study, GenBank accession numbers are shown in parenthesis.

### Tick RNA interference and capillary feeding

*D. variabilis *subolesin dsRNA and unrelated control Rs86 dsRNA were synthesized as described previously [8,15,32], using the Access RT-PCR system (Promega, Madison, WI, USA) and the Megascript RNAi kit (Ambion, Austin, TX, USA). The dsRNA was purified and quantified by spectrometry. Male *D. variabilis *ticks were injected with approximately 0.4 μl of dsRNA (5 × 10^10^-5 × 10^11 ^molecules per μl) in the lower right quadrant of the ventral surface of the exoskeleton of ticks [8,15]. The injections were done on 30 ticks per group using a Hamilton syringe with a 1-inch, 33 gauge needle. The ticks were held in a humidity chamber for 1 day after which they were allowed to feed for 3 days on a sheep prior to CF. Ticks were removed from the sheep and immobilized for CF [28]. Fifty-μl volume capillary tubes were placed over the capitulum of the ticks to feed them with the pathogen containing tick meal (Table 2). CF was done for 3 days with daily changes of capillary tubes containing fresh tick meal. Whole individual ticks were then dissected and used for DNA/RNA extraction to determine pathogen infection levels by real-time PCR and subolesin mRNA levels by real-time RT-PCR using pathogen-specific gene sequences (Table 3) and subolesin primers, respectively, as described above. Subolesin and pathogen-specific gene normalized Ct values were compared between subolesin dsRNA and control Rs86 dsRNA injected ticks by Student's t-test (P = 0.05). For *F. tularensis*, dissected tick tissues were homogenized, centrifuged and supernatants plated to count pathogen colony forming units (CFU) per tick and to compare CFU between subolesin dsRNA and control Rs86 dsRNA injected ticks by Student's t-test (P = 0.05). Regression analyses were conducted in Microsoft Excel to compare normalized pathogen infection levels and subolesin mRNA levels.

## Authors' contributions

JF conceived and designed the experiments. JF, AT, SC, KMK and FJ coordinated the experiments. KMK, ZZ, AT, RM, AA, SS, KMK, EB, MV, RCG, RIM, GLB and AMN performed the experiments. ZZ, AT, RM and JF analyzed the data. JF, KMK, ZZ, AT and RM wrote the paper. All the authors read and approved the final manuscript.
